# Effects of Hemagglutination Activity in the Serum of a Deep-Sea Vent Endemic Crab, *Shinkaia Crosnieri*, on Non-Symbiotic and Symbiotic Bacteria

**DOI:** 10.1264/jsme2.ME15066

**Published:** 2015-07-25

**Authors:** So Fujiyoshi, Hiroaki Tateno, Tomoo Watsuji, Hideyuki Yamaguchi, Daisuke Fukushima, Sayaka Mino, Makoto Sugimura, Tomoo Sawabe, Ken Takai, Shigeki Sawayama, Satoshi Nakagawa

**Affiliations:** 1 Laboratory of Marine Environmental Microbiology, Graduate School of Agriculture, Kyoto University, Oiwake-cho, Kyoto 606–8502, Japan; 2 Research Center for Stem Cell Engineering, National Institute of Advanced Industrial Science and Technology (AIST), Central 2, 1–1–1 Umezono, Ibaraki 305–8568, Japan; 3 Subsurface Geobiology and Advanced Research project (SUGAR), Institute of Biogeosciences, Japan Agency for Marine-Earth Science and Technology (JAMSTEC), 2–15 Natsushima-cho, Yokosuka 237–0061, Japan; 4 Leica Microsystems K.K. Osaka Sales Office ShogyoNo.2 Bldg. 10F, 5–4–9 Toyosaki, Kita-ku, Osaka 531–0072, Japan; 5 Laboratory of Microbiology, Faculty of Fisheries Sciences, Hokkaido University, 3–1–1 Minato-cho, Hakodate 041–8611, Japan; 6 Enoshima Aquarium, 2–19–1 Katase Kaigan, Fujisawa, Kanagawa 251–0035, Japan

**Keywords:** Hemagglutinin/lectin, Deep-sea hydrothermal field, Self/non-self recognition, Host-microbe interaction, Symbiosis

## Abstract

In deep-sea hydrothermal environments, most invertebrates associate with dense populations of symbiotic microorganisms in order to obtain nutrition. The molecular interactions between deep-sea animals and environmental microbes, including their symbionts, have not yet been elucidated in detail. Hemagglutinins/lectins, which are carbohydrate-binding proteins, have recently been reported to play important roles in a wide array of biological processes, including the recognition and control of non-self materials. We herein assessed hemagglutination activity in the serum of a deep-sea vent endemic crab, *Shinkaia crosnieri*, which harbors chemosynthetic epibionts on its plumose setae. Horse and rabbit erythrocytes were agglutinated using this serum (opt. pH 7.5 and opt. temperature 15°C). Agglutinating activity was inhibited by eight kinds of sugars and several divalent cations, did not require any divalent metal ions, and remained detectable even after heating the serum at 100°C for 30 min. By using fluorescently labeled serum, we demonstrated that deep-sea crab serum components bound to the epibionts even in the presence of sugars. This study represents the first immunological assessment of a deep-sea vent endemic crab and demonstrated the possibility of a non-lectin-mediated symbiont-host interaction.

Deep-sea hydrothermal vents host highly productive ecosystems fueled primarily by chemosynthetic microbial production. Most invertebrates thrive in this extraordinary environment through their association with chemosynthetic symbionts. This chemosynthetic association is morphologically diverse, ranging from loose episymbiosis to obligate endosymbiosis (7). In siboglinid tubeworms, a sulfur- and potentially hydrogen-oxidizing gammaproteobacterial symbiont is housed in the interior of the worm in a specialized organ, the trophosome (3, 28). Many deep-sea vent endemic mollusks, such as mytilid mussels, vesicomyid clams, and some provannid gastropods harbor their endosymbionts in enlarged gills (7). Scaly-foot gastropods, which are snails with scales that cover the sides of its foot, have gammaproteobacterial endosymbionts in their enlarged esophageal glands (8, 24). In contrast, decapod crustaceans typically harbor a diverse range of episymbiotic chemolithoautotrophs and methanotrophs (9, 29). In most deep-sea endemic animals, symbionts are taken up from the environment anew by each host generation (4). Pathogen outbreaks have rarely been detected in deep-sea vent ecosystems, which may partly be due to the lack of studies on pathogens in these locations (35, 38). As a consequence, deep-sea vent endemic invertebrates need to respond accordingly to associations with various microbes including symbionts and pathogens; however, information on their immunological abilities currently remains limited.

Invertebrates, which lack adaptive immune systems, generally rely on innate immunity to respond to non-self materials. Cellular and humoral immune components are mainly found in the hemolymph. Pattern recognition proteins (PRPs) or pattern recognition receptors (PRRs) recognize and bind evolutionarily conserved pathogen-associated molecular patterns (PAMPs) on the surface of invading microbes, such as lipopolysaccharide (LPS) from Gram-negative bacteria, peptidoglycans and lipoteichoic acid (LTA) from Gram-positive bacteria, mannans from fungi, and glycoproteins from viruses (33). Among crustacean innate immune components, hemolymphatic agglutinins (lectins) are known to play important roles as a sensor and regulator of foreign organisms (17). Lectins are glycan-binding proteins or glycoproteins that recognize the cell surface glycoconjugates of microorganisms and then induce various immune responses, such as immobilization, phagocytosis, clearance, and encapsulation (17). The lectins of marine animals have recently been extensively screened and characterized because of their therapeutic and industrial potentials. Many marine animals have lectins that belong to the C-type lectin family, which bind to glycans in a Ca^2+^-dependent manner (27). Lectins have also been reported to play an important role in symbiosis. Symbiosis-related lectins have been detected in coral-zooxanthellae and Hawaiian bobtail squid-*Vibrio* symbiosis (12, 18).

In order to address host-microbe interactions in deep-sea environments, we previously analyzed the serum of the deep- sea vent endemic galatheid crab, *Shinkaia crosnieri*, which represents one of the dominant macrofauna in deep-sea hydrothermal fields in the Okinawa Trough, Japan (2). These crabs aggregate close to hydrothermal fluid flows (in which the average habitat temperature is <10°C) and harbor dense populations of chemolithoautotrophic and methanotrophic epibionts attached to their plumose setae. The epibionts mainly consist of sulfur-oxidizing *Epsilonproteobacteria* and sulfur- or methane-oxidizing *Gammaproteobacteria* (36, 39). Many epibiotic *Epsilonproteobacteria* exhibit an extremely thick (up to 5.5 μm in diameter), segmented, and filamentous morphology (13, 39). Epibionts with a similar morphology have also been reported in the deep-sea vent endemic crustaceans, *Kiwa* and *Rimicaris* species (9, 29). Close phylogenetic relatives of the epibiotic and filamentous *Epsilonproteobacteria* of *S. crosnieri* have been detected in and cultured from the crab habitat and its surroundings (20–22); however, a thick and filamentous morphology has never been observed in laboratory enrichment cultures and isolates, thereby suggesting certain biological interactions for selection and/or morpho-physiological control between the epibionts and host animal.

We herein demonstrated for the first time the presence of lectins in the serum of *S. crosnieri* and serum component(s) binding to its epibionts. We also found that binding to epibionts was not inhibited by sugars, which suggests that the association between *S. crosnieri* and its epibionts may serve as a novel system that differs from the model symbiotic systems identified to date.

## Materials and Methods

### Collection of *S. crosnieri* from a deep-sea vent field

**Fig. 1:**
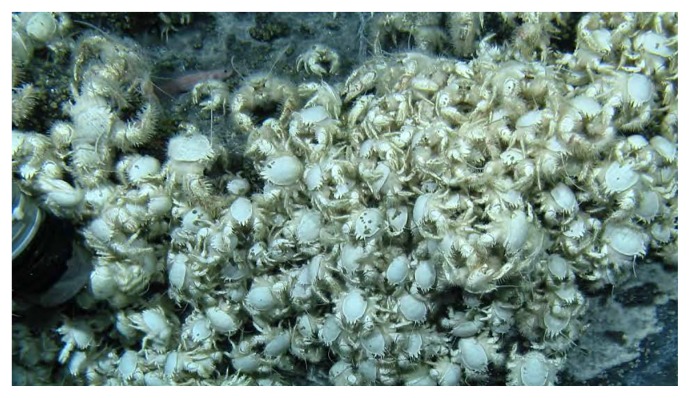
Sampling *Shinkaia crosnieri* at the top of a sulfide mound in the Iheya North hydrothermal field.

Individuals of *S. crosnieri* were collected from the Iheya North hydrothermal field (27°47.46′ N, 126°53.80′ E; water depth of approximately 1,000 m) in the Okinawa Trough, Japan, by means of a remotely operated vehicle (ROV), “*Hyper-Dolphin*” of the Japan Agency for Marine-Earth Science and Technology (JAMSTEC) (dives #1324–1326 on September 24–27, 2011; Fig. 1). Most of the crabs collected were still alive after pressure and temperature changes during recovery. Once onboard the ship, the membrane between the carapace and abdomen was cut, and the hemolymph was collected on ice using a 1,000 μL pipet and then stored at −30°C. Episymbiotic bacterial cells, together with setae, were collected with flame-sterilized scissors. The hemolymph was centrifuged (12,000×*g* for 10 min at 4°C), and the supernatant serum was used for further analyses.

### Ion chromatography

The serum was diluted 2,000-fold and analyzed for its ionic composition by ion chromatography. Cations (Na^+^, K^+^, Mg^2+^, Ca^2+^) and anions (Cl^−^, Br^−^, NO_3_^−^, SO_4_^2−^, PO_4_^2−^) were quantified with a Dionex ion chromatography system (ICS-1600 equipped with Ion Pac CS12A and ICS-2100 equipped with an Ion Pac AS11-HC column, respectively; Dionex Corp., Sunnyvale, CA, USA).

### Preparation of erythrocytes for the hemagglutination (HA) activity test

Horse, rabbit, cow, and sheep blood was purchased from Kohjin Bio (Saitama, Japan). Erythrocytes were harvested and washed three times in a Bis-Tris buffer (25 mM Bis-Tris (hydroxymethyl aminomethane)-HCl, pH 7.5, 0.5 M NaCl) through centrifugation (2,000×*g* for 5 min at room temperature). Horse, rabbit, cow, and sheep erythrocytes were treated with trypsin (0.01% [w/v]) for 1 h at 37°C, and then washed three times with Bis-Tris buffer. Horse, cow, and sheep erythrocytes were fixed with formalin for longer storage (26), while rabbit erythrocytes were fixed with glutaraldehyde (37).

### HA activity measurement

HA activity was determined in V-bottom microtiter plates. A series of serial two-fold dilutions of crab serum were prepared using Bis-Tris buffer. An equal volume of a 1% (v/v) erythrocyte suspension in Bis-Tris buffer was added to each well. The plates were incubated for 2 h at 15°C. HA activity was expressed as the inverse of the last dilution showing visible hemagglutination. The control consisted of the substitution of the serum sample by Bis-Tris buffer.

### Cross-absorption test

Thirty microliters of *S. crosnieri* serum was absorbed with the same volume of horse or rabbit erythrocytes for 2 h at 15°C. After centrifugation (500×*g* for 5 min at 15°C), the supernatant of each absorbed serum was tested for hemagglutinating activity against horse or rabbit erythrocytes as described above.

### Inhibition of HA

The carbohydrate-binding spectrum of the serum was assessed by the ability of sugars and glycoproteins to inhibit hemagglutination. The HA inhibition test was performed in a similar manner to the HA activity test as described above. Ten microliters of 0.5 M sugar (D-glucosamine [GlcN], D-mannose [Man], L-arabinose [Ara], D-glucose [Glc], D-galactose [Gal], D-fructose [Fru], D-xylose [Xyl], *N*-acetylglucosamine [GlcNAc], *N*-acetylgalactosamine [GalNAc], *N*-acetylmannosamine [ManNAc], *N*-acetylneuraminic acid [NeuAc], lactose [Lac], maltose [Mal], or melibiose [Meli]) was diluted in Bis-Tris buffer with two-fold serial dilutions. A similar dilution procedure was applied to 2.0 mg mL^−1^ of glycoproteins (fetuin or bovine submaxillary mucin [BSM]). The serum sample was diluted in Bis-Tris buffer to 128 HA activity units (HU) for horse erythrocytes and 4 HU for rabbit erythrocytes, 10 μL of which was added to each well and incubated for 60 min at 15°C. Twenty microliters of the 1% (v/v) trypsin-untreated horse or rabbit erythrocyte suspension in Bis-Tris buffer was then added. The lowest concentration of a specific sugar or glycoprotein that inhibited hemagglutination was defined as the minimum inhibitory concentration (MIC).

### Microarray analysis

In addition to traditional HA-dependent methods, a glycoconjugate microarray with an evanescent-field fluorescence detection system was used to assess the glycan-binding specificity of serum components (34). Serum components were fluorescently labeled using a Cy3 Mono-reactive Dye kit (GE healthcare, Buckinghamshire, UK), and dissolved at a concentration of 5% (v/v) in a buffer (25 mM Tris-HCl, pH 7.4 containing 0.8% [w/v] NaCl, 1% [v/v] Triton-X, 1 mM MnCl_2_, 1 mM CaCl_2_). The labeled serum was applied to a glycoconjugate microarray. After overnight incubation at 20°C, the unbound proteins were washed out. Fluorescent images were scanned by an evanescent-field activated fluorescence scanner (GlycoStation Reader 1200; Glycotechnica, Sapporo, Japan) under the Cy3 mode and analyzed with the Array Pro analyzer ver. 4.5 (Media Cybernetics, Rockville, MD, USA). The glycans used for the glycoconjugate microarray were shown in Table S1.

### Effects of pH, temperature, and divalent cations on HA activity

The pH-dependency of HA was evaluated using horse or rabbit erythrocytes. The serum was concentrated by ultrafiltration (MWCO= 10,000 Da), dissolved, and serially diluted in the following buffers (25 mM and all contained 0.5 M NaCl): sodium acetate (pH 4.0), Bis-Tris (pH 6.5–7.5), Tris-HCl (pH 8.5), and sodium carbonate (pH 10.0). Ten microliters of the serum solutions were respectively mixed with 10 μL of the 1% (v/v) trypsin-untreated horse or rabbit erythrocyte suspension in the corresponding buffer. Following overnight incubation at 4°C, HA activity was determined. In addition, the heat stability of HA activity was examined using the serum incubated for 30 min at various temperatures (40, 50, 60, 70, 80, 90, and 100°C). After cooling at 4°C, residual HA activity was measured against the 1% trypsin-untreated horse or rabbit erythrocyte suspension as described above. In order to determine the effects of divalent cations or EDTA on HA activity, HA activity was measured in the presence of various concentrations of CaCl_2_·2H_2_O (1, 5, 10, 20, and 30 mM), 10 mM MgCl_2_·6H_2_O, 10 mM MnCl_2_·4H_2_O, or 10 mM EDTA.

### Association of serum components with bacterial cells

Serum components were fluorescently labeled as described above, and dissolved in Bis-Tris buffer (2% [v/v]). The solution was incubated for 6 h at 15°C with the washed bacterial cells of *Escherichia coli* Es1, *Bacillus subtilis* B7, deep-sea vent *Epsilonproteobacteria* (*Sulfurovum* sp. NBC 37-1 and *Sulfurimonas autotrophica* OK10) (10, 23), and epibiotic bacterial cells physically separated from *S. crosnieri*. Microbial cells were counter-stained with 4′,6-diamidino-2-phenylindole (DAPI) and observed with a fluorescence microscope (Axio Imager2 upright microscope, Carl ZEISS) or confocal laser scanning microscope (TCS-SP5, Leica). The agglutination of bacteria by the serum was also assayed in the presence of sugars (0.5 M Man, 0.25 M Lac). After being incubated with the serum (10 μL) and each sugar (10 μL) in 100 μL of buffer (25 mM Bis-Tris, 0.5 M NaCl, pH 7.5) for 6 h at 15°C, cells were observed with the microscope. Bovine serum albumin (BSA) (1 mg mL^−1^) was substituted for carbohydrates in assay controls.

### Effects of the serum on the growth of bacteria

We assessed the growth of culturable mesophilic *Epsilonproteobacteria*, *i.e. Sulfurovum* sp. NBC37-1 and *S. autotrophica* OK10, in the presence or absence of the serum. The isolates were grown in 3 mL of MMJHS medium (32) containing 200 or 500 μL of serum at 15°C. Growth was monitored in triplicate by the direct counting of DAPI-stained cells under the microscope.

## Results and Discussion

### HA assay, HA inhibition assay, and microarray analysis

**Fig. 2:**
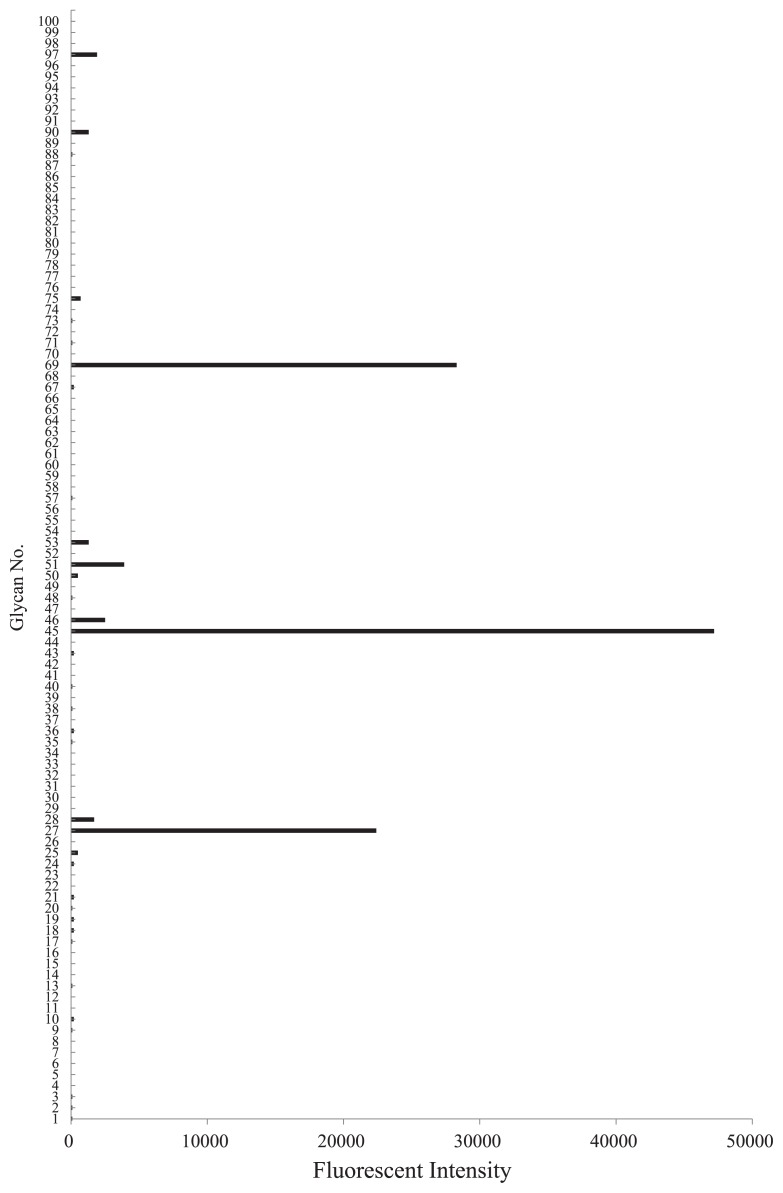
Fluorescent signals showing the reaction of *S. crosnieri* serum with various glycoconjugates (1–100). Detailed information on the glycans used in this study can be found in Table S1.

The serum of *S. crosnieri* agglutinated trypsin-treated and -untreated horse erythrocytes and rabbit erythrocytes at 15°C. The strongest agglutination titer was towards horse erythrocytes (Table 1). The trypsin treatment reduced HA for rabbit erythrocytes, but not for horse erythrocytes. The HA activity of the serum against rabbit erythrocytes was partially absorbed with horse erythrocytes, whereas that against horse erythrocytes was not absorbed with rabbit erythrocytes (Table 2). This result suggested the presence of multiple lectins in the serum. The HA of horse and rabbit erythrocytes has been reported in many glucose- and mannose-binding lectins from plants, including concanavalin A (ConA), lentil lectin (LCL), and pea lectin (PSL) (15). Sugar-binding specificity was examined using HA inhibition assays with various carbohydrates and glycoproteins. Four sugars (GlcN, Man, Mal, and Lac) inhibited the HA activity of the serum against horse erythrocytes, while 6 sugars (GlcN, Ara, Gal, GalNAc, Lac, and Meli) inhibited that against rabbit erythrocytes (Table 3). Although many previously characterized crustacean lectins appear to share a common binding specificity to NeuAc (17), an evanescent-based glycoconjugate microarray analysis confirmed the absence of specific activity to NeuAc (Fig. 2). *S. crosnieri* serum exhibited more binding activity towards asialo-glycophorin (No. 69) than glycophorin (No. 75), suggesting that the serum recognized asialo *O*-glycans, most likely the core1 structure (Galβ1-3GalNAc) (1) (Fig. 2). The serum also displayed binding activity to native, asialo, and agalacto transferrin (No. 27, 45, and 51, respectively). However, this binding may have been protein-protein interactions because the serum bound to all types of transferrin with different glycan structures. Taken together with the results obtained by the hemagglutination inhibition assay, *S. crosnieri* serum may contain lectins with specificity to galactose-related sugars.

### Effects of pH, temperature, and divalent cations on hemagglutination activity

**Fig. 3:**
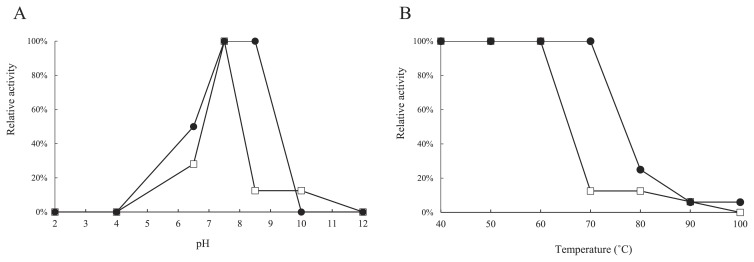
Effects of pH (A) and temperature (B) on the HA activity of *S. crosnieri* serum against horse erythrocytes (●) and rabbit erythrocytes (□).

The HA activity of *S. crosnieri* serum was observed within a narrow pH range (pH 6.5–8.5, opt. pH 7.5–8.5 [assessed with horse erythrocytes]; pH 6.5–10, opt. pH 7.5 [assessed with rabbit erythrocytes]) (Fig. 3A). The pH of sampled *S. crosnieri* serum was 6.8–7.2. HA activity was highly thermostable because it remained 100% active up to 70°C for 30 min and was detectable even after heating at 100°C for 30 min against horse erythrocytes (Fig. 3B). The *S. crosnieri* population dwells in widespread benthic habitats in which hydrothermal fluids mix with ambient seawater (39). This habitat is generally characterized by a low temperature (similar to the ambient seawater temperature of 4°C), but is occasionally affected by extremely high-temperature (over 300°C) fluids. This may be relevant for the high thermostability of HA activity. Nevertheless, markedly thermostable lectins have been reported from the rhizome of *Kaempferia parviflora* (14) and the mushroom of *Ganoderma capense* (25). Furthermore, slightly thermostable lectins have been reported from the insect *Geotrupes stercorarius* (6) and the marine invertebrate *Holothuria grisea* (19). Thus, relatively high thermostability may be largely derived from the primary structure common to various lectins.

HA activity was not affected by EDTA, but was markedly inhibited by 30 mM Ca^2+^ and 10 mM Mn^2+^ (Table 4). Many lectins of marine invertebrates are reversibly or non-reversibly sensitive to EDTA and require Ca^2+^ for their activity (27). Some shrimp lectins have been purified and characterized in buffers containing 10 mM Ca^2+^ (16, 30, 31). Likewise, the lectin of *Calyptogena okutanii*, a deep-sea vent bivalve, exhibited maximum activity at 10 mM Ca^2+^ (a similar level to the concentration of seawater) (11). The serum ionic composition of *S. crosnieri* was determined as follows: 453.2 mM Cl^−^, 24.3 mM SO_4_^2−^, 1.1 mM PO_4_^2−^, 0.7 mM Br^−^, 0.01 mM NO_3_^−^, 421.1 mM Na^+^, 35.5 mM Mg^2+^, 12.0 mM Ca^2+^, and 8.7 mM K^+^. The abundance of divalent cations in the serum may repress glycan-binding activity *in vivo*. Significant fluctuations in serum ionic components that are dependent on the molting cycle have been reported in crustaceans (41). Although molting has never been observed in *S. crosnieri*, HA activity detected in the serum may play a role in interactions with environmental bacteria including epibionts at the postmolt stage. Time-course analyses of *S. crosnieri* individuals during their life history may assist in defining the role of unique HA activity in the serum; however, extreme difficulties are still associated with the long-term rearing of deep-sea animals.

### Association of serum components with bacterial cells

**Fig. 4:**
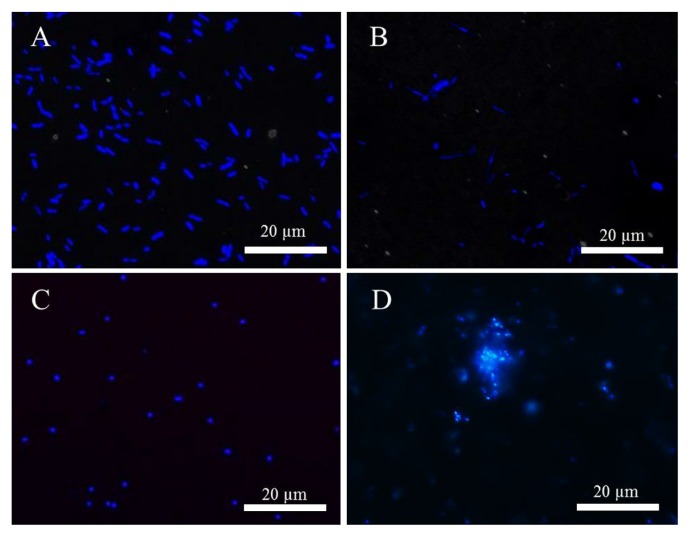
Association of serum components with bacterial cells. Cells were reacted with crab serum, and labeled with DAPI (blue). (A) *E. coli* Es1, (B) *B. subtilis* B7, (C) *Sulfurovum* sp. NBC 37-1, and (D) *S. autotrophica* OK10.

**Fig. 5:**
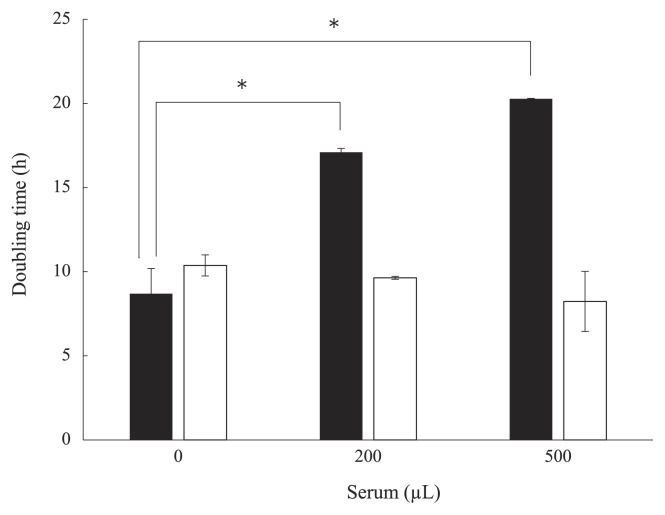
Effects of the serum on bacterial growth. *S. autotrophica* OK10 (black bars), *Sulfurovum* sp. NBC37-1 (white bars). Statistical comparisons were performed by the Student’s *t*-test (*n*=3, *p<0.05).

Lectins from invertebrates are mainly known as immunomodulators that are active against Gram-negative and Gram-positive bacteria. A lectin that preferentially bound to Gram-negative bacteria was detected in shrimp *Litopenaeus vannamei* (30). Furthermore, a lectin with the ability to bind Gram-negative and -positive bacteria was identified in shrimp *Penaeus japonicas* (40). However, as shown in Fig. 4 (A and B), the serum of *S. crosnieri* did not bind to *E. coli* (Gram-negative bacteria) or *B. subtilis* (Gram-positive bacteria). The serum did not bind to the cells of *Sulfurovum* sp. NBC37-1 (Fig. 4C) and did not affect its growth (Fig 5) in spite of *Sulfurovum* representing the predominant population in the epibiotic bacterial community of *S. crosnieri* (36, 39). In contrast, *S. autotrophica* OK10, isolated from the Hatoma Knoll in the Mid-Okinawa Trough hydrothermal field (10), was aggregated by the serum of *S. crosnieri* (Fig. 4D). This aggregation was inhibited by Lac (Fig. S1), suggesting that aggregation was caused by the serum lectin(s). In addition, the growth of *S. autotrophica* OK10 was inhibited by the serum (Fig. 5). This result suggests that the serum has the ability to control some non-symbiotic bacterial growth.

**Fig. 6:**
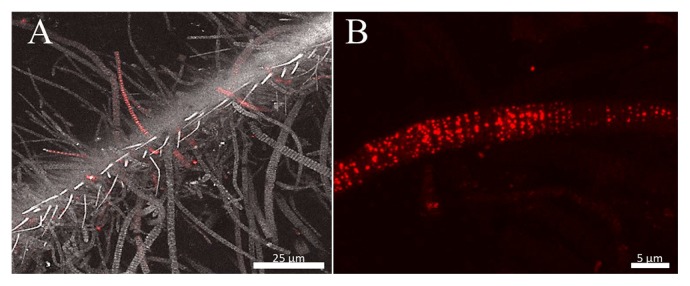
Confocal laser scanning microscopy of filamentous episymbiotic bacteria reacted with Cy3 labeled serum components of *S. crosnieri* (red). (A) Light and fluorescent images were merged. Filamentous bacteria were selectively stained. (B) Magnified view of a filamentous bacterium.

The lectin from octocoral *Sinularia lochmodes* not only binds to the symbiotic algae *Symbiodinium*, but is also known to be toxic to some non-symbiotic algae (13). In a marine nematode, the C-type lectin of the host was found to mediate the initial interaction between the host and episymbiont, followed by a tighter and irreversible binding (4, 5). These symbiotic models suggest the important role of lectin in host-symbiont interactions. We herein found that fluorescently (Cy3) labeled serum components specifically bound to some filamentous epibionts in a spotty manner (Fig. 6). However, the Cy3 signal was observed even under the presence of lectin inhibitory sugars (Fig. S2). This result reflected filamentous epibionts having specific structures on their cell surface, and crab serum containing some proteinaceous, but not lectin components for the recognition of epibiont cells under certain physiological states.

## Conclusion

Even though over 35 years have passed since the discovery of deep-sea vent ecosystems, the molecular basis of symbiotic interactions between host animals and chemosynthetic microbial symbionts has not yet been elucidated. In the last decade, some close relatives of symbiotic bacteria have been obtained in pure cultures and sophisticated rearing methods for deep-sea animals have been developed for some chemosynthetic animal species. Furthermore, omics analyses have recently provided an unprecedented abundance of information on the evolution, physiology, and ecology of deep-sea vent microorganisms and even chemosynthetic animals. In the present study, we demonstrated the unique HA activity and antimicrobial activity of some non-symbiotic bacteria in *S. crosnieri*. This study represents the first immunological assessment of a deep-sea vent endemic crab and shows the possibility of a non-lectin mediated symbiont-host interaction. In addition to ongoing efforts using cultivation and omics techniques, further immunological or glycobiological characterizations will be necessary for future studies on deepsea hydrothermal vent symbiosis.

## Supplementary Information



## Figures and Tables

**Table 1:** HA against different erythrocytes

Types of erythrocytes	HA titer (HU)
Horse	512
Horse (trypsin-treated)	512
Rabbit	32
Rabbit (trypsin-treated)	16
Cow	ND
Cow (trypsin-treated)	ND
Sheep	ND
Sheep (trypsin-treated)	ND

* ND, HA not detected.

**Table 2:** HA activity of *S. crosnieri* serum before and after absorption with several erythrocytes

Absorbed with:	HA titer (HU)	

	Type of erythrocytes	

	Horse	Rabbit
Horse	ND	ND
Rabbit	16	ND
None	512	32

* ND, HA not detected.

**Table 3:** HA inhibition of the serum by various sugars and glycoproteins against horse or rabbit erythrocytes

	Minimum inhibitory concentration (mM)	
	
	Horse erythrocytes	Rabbit erythrocytes
GlcN	31.2	15.6
Man	125	NI
Ara	NI	31.2
Glc	NI	NI
Gal	NI	62.5
Fru	NI	NI
Xyl	NI	NI
GlcNAc	NI	NI
GalNAc	NI	3.9
ManNAc	NI	NI
NeuAc	NI	NI
Mal	15.6	NI
Lac	7.8	7.8
Meli	NI	15.6
Fetuin	NI	NI
BSM	NI	NI

* NI: No inhibition at 200 mM of sugar or 2 mg mL^−1^ of glycoprotein.

**Table 4:** Effects of EDTA and divalent cations on the HA activity of *S. crosnieri* serum against horse or rabbit erythrocytes

	EDTA	Ca^2+^					Mn^2+^	Mg^2+^
				
Conc. (mM)	10	1	5	10	20	30	10	10
HA titer (HU)								
Horse erythrocytes	512	512	512	2	ND	ND	2	32
Rabbit erythrocytes	32	16	64	64	16	ND	ND	128
