# Supplementary material for: Effects of Hemagglutination Activity in the Serum of a Deep-Sea Vent Endemic Crab, Shinkaia Crosnieri, on Non-Symbiotic and Symbiotic Bacteria
Source: Microbes Environ. 2015 Jul 25;30(3):228–34. doi: 10.1264/jsme2.ME15066 (PMC4567561; doi:10.1264/jsme2.ME15066)
Supplement: Supplementary file 1 [file 30_228_s1.pdf]

Supplementary data.

Table S1. Glycans used for glycoconjugate microarray.

Number	Glycans
1	Fuca1-PAA
2	Fuca1-2Galb1-PAA
3	Fuca1-3GlcNAcb1-PAA
4	Fuca1-4GlcNAcb1-PAA
5	Fuca1-2Galb1-3GlcNAcb1-PAA
6	Fuca1-2Galb1-4GlcNAcb1-PAA
7	Fuca1-2Galb1-3GalNAca1-PAA
8	GalNAca1-3(Fuca1-2)Galb1-4GlcNAcb1-PAA
9	Gala1-3(Fuca1-2)Galb1-4GlcNAcb1-PAA
10	Galb1-3(Fuca1-4)GlcNAcb1-PAA
11	(3OSO$_3$)Galb1-3(Fuca1-4)GlcNAcb1-PAA
12	Fuca1-2Galb1-3(Fuca1-4)GlcNAcb1-PAA
13	Galb1-4(Fuca1-3)GlcNAcb1-PAA
14	Fuca1-2Galb1-4(Fuca1-3)GlcNAcb1-PAA
15	Neu5Aca2-PAA
16	Neu5Gca2-PAA
17	Neu5Aca2-8Neu5Aca2-PAA
18	Neu5Aca2-8Neu5Aca2-8Neu5Aca2-PAA
19	Neu5Aca2-3Galb1-3GlcNAcb1-PAA
20	Neu5Aca2-3Galb1-4Glcb1-PAA
21	Neu5Aca2-3Galb1-4GlcNAcb1-PAA
22	Neu5Aca2-3Galb1-3(Fuca1-4)GlcNAcb1-PAA
23	Neu5Aca2-3Galb1-4(Fuca1-3)GlcNAcb1-PAA
24	Neu5Aca2-6Galb1-4Glcb1-PAA
25	Fetuin (Complex-type N-glycans and O-glycans)
26	a1-acid glycoprotein (Complex-type N-glycans-)
27	Transferrin (Complex-type N-glycans)
28	Porcine thyroglobulin (Complex and high-mannnose-type N-glycans)
29	Galb1-PAA
30	(3OSO$_3$)Galb1-PAA
31	GalNAca1-3Galb1-PAA
32	GalNAca1-3Galb1-

33	Galb1-3GlcNAcb1-PAA
34	$(3OSO_3)$Galb1-3GlcNAcb1-PAA
35	Galb1-4GlcNAcb1-PAA
36	$(3OSO_3)$Galb1-4GlcNAcb1-PAA
37	Galb1-4$(6OSO_3)$GlcNAcb1-PAA
38	$(6OSO_3)$Galb1-4GlcNAcb1-PAA
39	GalNAcb1-PAA
40	GalNAcb1-3GalNAcb1-PAA
41	GalNAcb1-4GlcNAcb1-PAA
42	GalNAcb1-4Galb1-4Glcb1-PAA
43	Asialo fetuin (Desialylated complex-type N- and O-glycans)
44	Asialo a1-acid glycoprotein (Desialylated complex-type N-glycans)
45	Asialo transferrin (Desialylated complex-type N-glycans)
46	Asialo porcine thyroglobulin (Desialylated complex-type N-glycans, high-mannose-type N-glycans)
47	GlcNAcb1-PAA
48	$(6OSO_3)$GlcNAcb1-PAA
49	Agalacto fetuin (Agalactosylated complex-type N- and O-glycans)
50	Agalacto a1-acid glycoprotein (Agalactosylated complex-type N-glycans)
51	Agalacto transferrin (Agalactosylated complex-type N-glycans, high-mannose-type N-glycans)
52	Ovomucoid (Complex-type N-glycans)
53	Ovoalbumin (Hybrid-type N-glycans)
54	Mana1-PAA
55	Manb1-PAA
56	$(6OPO_4)$Mana1-PAA
57	Yeast invertase (High mannose-type N-glycans)
58	GalNAca1-PAA
59	Galb1-3GalNAca1-PAA
60	Galb1-3(GlcNAcb1-6)GalNAca1-PAA
61	GlcNAcb1-3GalNAca1-PAA
62	GlcNAcb1-3(GlcNAcb1-6)GalNAca1-PAA
63	GalNAca1-3GalNAcb1-PAA
64	GlcNAcb1-6GalNAca1-PAA
65	Gala1-3GalNAca1-PAA

66	(3OSO$_3$)Galb1-3GalNAca1-PAA
67	Galb1-4GlcNAcb1-3GalNAca1-PAA
68	Asialo bovine submaxillary mucin (Tn)
69	Asialo human glycophorin MN (T)
70	Neu5Aca2-6GalNAca1-PAA
71	Neu5Gca2-6GalNAca1-PAA
72	Neu5Aca2-3Galb1-3GalNAca1-PAA
73	Galb1-3(Neu5Aca2-6)GalNAca1-PAA
74	Bovine submaxillary mucin (Sialyl Tn)
75	Human glycophorin (Disialyl T and sialyl Tn)
76	Gala1-PAA
77	Gala1-2Galb1-PAA
78	Gala1-3Galb1-PAA
79	Gala1-3Galb1-4Glcb1-PAA
80	Gala1-3Galb1-4GlcNAcb1-PAA
81	Gala1-4Galb1-4GlcNAcb1-PAA
82	Gala1-6Glcb1-PAA
83	Glca1-PAA
84	Glcb1-PAA
85	Glca1-4Glcb1-PAA
86	Hyaluronic acid-BSA
87	Chondoroitin Sulfate A-BSA
88	Chondoroitin Sulfate B-BSA
89	Heparan Sulfate-BSA
90	Heparin-BSA
91	Keratan Sulfate-BSA
92	Rhamnosea1-PAA
93	*S. cerevisiae* mannan
94	*C.albicans* mannan
95	Zymosan
96	GlcNAcb1-4GlcNAcb1-PAA
97	Bovine serum albumin
98	Negative PAA
99	Marker
100	Back ground

BSA: bovine serum albmin, PAA: polyacrylamide

Supplementary figure legends

Fig. S1. Effects of sugar on the serum agglutination activity against *S. autotrophica* OK10. The bacterial cells were incubated with the following: (A) control (cells were incubated with the serum and BSA (1.0 mg ml^{-1}), (B) negative control (cells were incubated with a buffer: 25 mM Bis-Tris, 0.5 M NaCl, pH 7.5), (C) the serum and GlcN (0.5 M), (D) GlcN (0.5 M), (E) the serum and Lac (0.25 M), (F) Lac (0.25 M).

Fig. S2. Confocal laser scanning microscopy of filamentous episymbiotic bacteria reacted with Cy-3 labeled serum components (red) of *S. crosnieri*. Light and fluorescent images were merged. The serum and episymbiotic bacterial cells were incubated with the follo

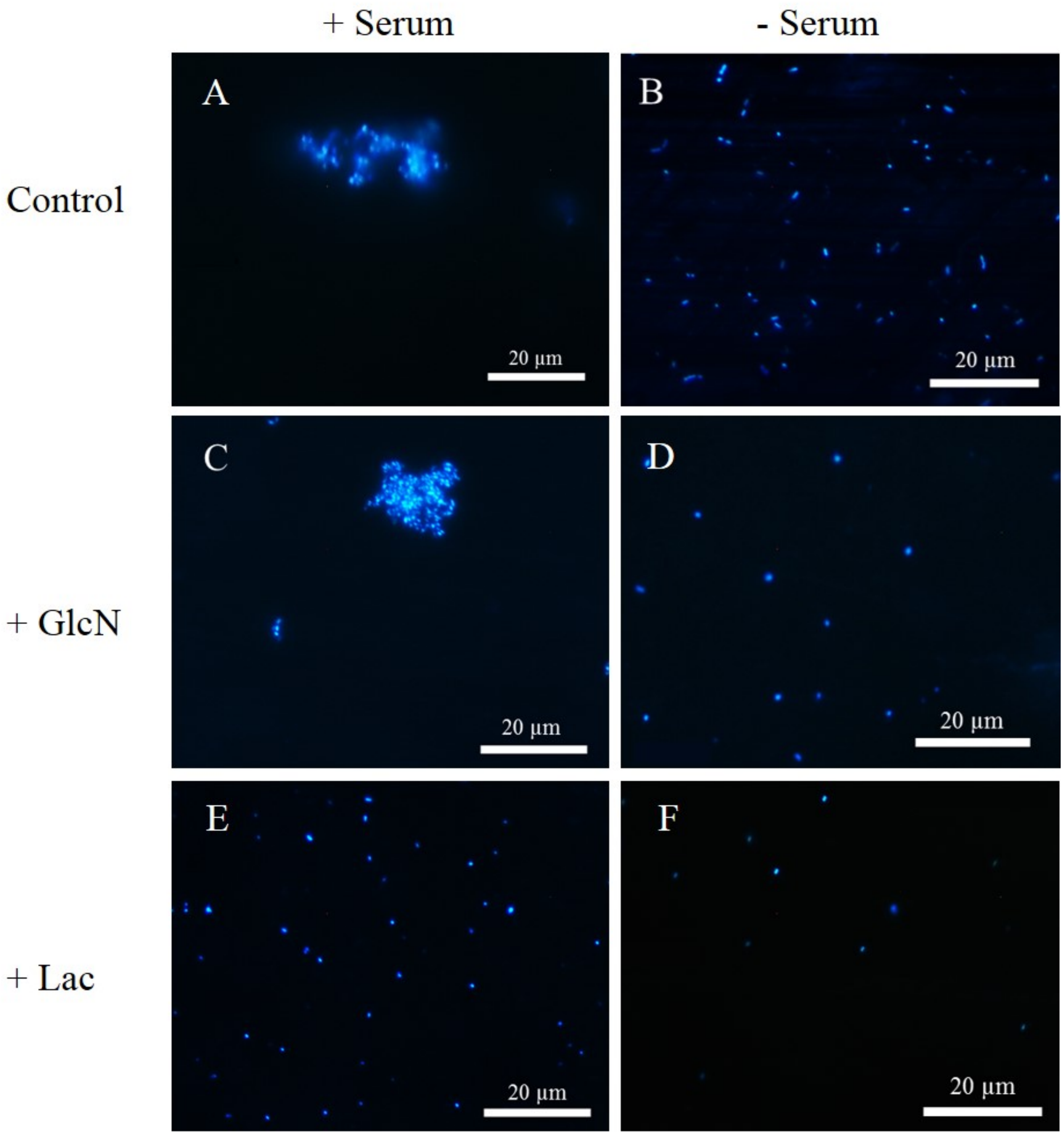

Fig. S1 So Fujiyoshi

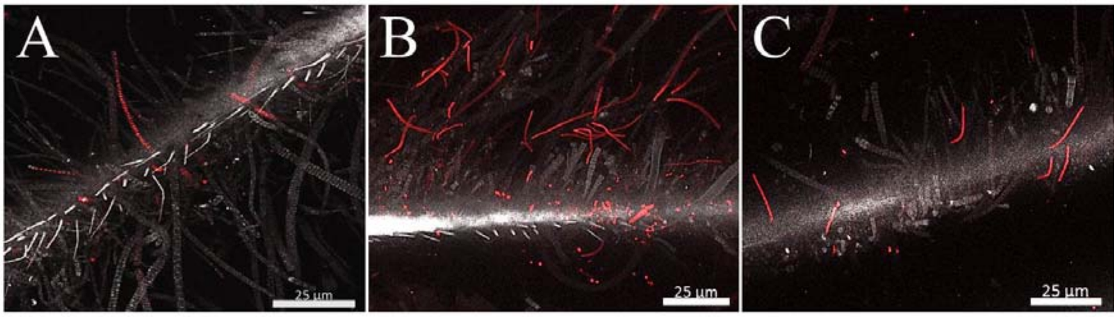

Fig. S2 So Fujiyoshi
